# Pregnancy‐associated osteoporosis following in vitro fertilization: A case report

**DOI:** 10.1002/ccr3.8702

**Published:** 2024-03-21

**Authors:** Mehran Rahimi, Sara Daneshvar, Alireza Khabbazi

**Affiliations:** ^1^ Connective Tissue Diseases Research Center Tabriz University of Medical Sciences Tabriz Iran

**Keywords:** case report, gonadotropin‐releasing hormone, in vitro fertilization (IVF), ovarian aging, pregnancy and lactation‐associated osteoporosis, pregnancy‐associated osteoporosis

## Abstract

This case report illustrates that in vitro fertilization (IVF) may be a potential risk factor for pregnancy‐associated osteoporosis (PAO), highlighting the need for awareness and monitoring of bone health in women undergoing IVF treatments. PAO is a rare disease resulting from an imbalance of calcium in the body during pregnancy and lactation and presenting with fragility fractures. PAO occurs in late pregnancy or early postpartum period. A 28‐year‐old woman who conceived through IVF experienced severe back pain 2 days after delivery. Magnetic resonance imaging of the spine showed wedge‐shaped fractures of T9‐T12 vertebrae. Bone mineral density (BMD) was low on dual‐energy x‐ray absorptiometry. The laboratory tests were within the normal range. Based on the clinical manifestations, osteoporotic spine fracture, results of BMD, and exclusion of other causes of osteoporosis, the patient was diagnosed with PAO. Considering the deleterious effect of treatment with gonadotropin‐releasing hormone and repeated superovulation on bone, we hypothesized that IVF may be an etiological factor for PAO.

## 1 INTRODUCTION

Pregnancy‐associated osteoporosis (PAO) is a rare disorder that occurs as a result of an imbalance of calcium in the body during pregnancy and lactation and presents with low bone mineral density (BMD) and fragility fractures in the spine and rarely in other bones. Although the underlying pathological factor responsible for PAO is unknown, it has been suggested that PAO is a multifactorial disease.^1^ Several risk factors have been identified for this disease, including a family history of osteoporosis or fracture, history of anorexia nervosa, smoking, dental problems during childhood, low physical activity, body mass index (BMI) <18 kg/m^2^, hypovitaminosis D, immobilization during pregnancy and heparin use during pregnancy.^1^, ^2^ Here, we report a case of PAO in a woman conceived by in vitro fertilization (IVF).

## 2 CASE HISTORY/EXAMINATION

**FIGURE 1:**
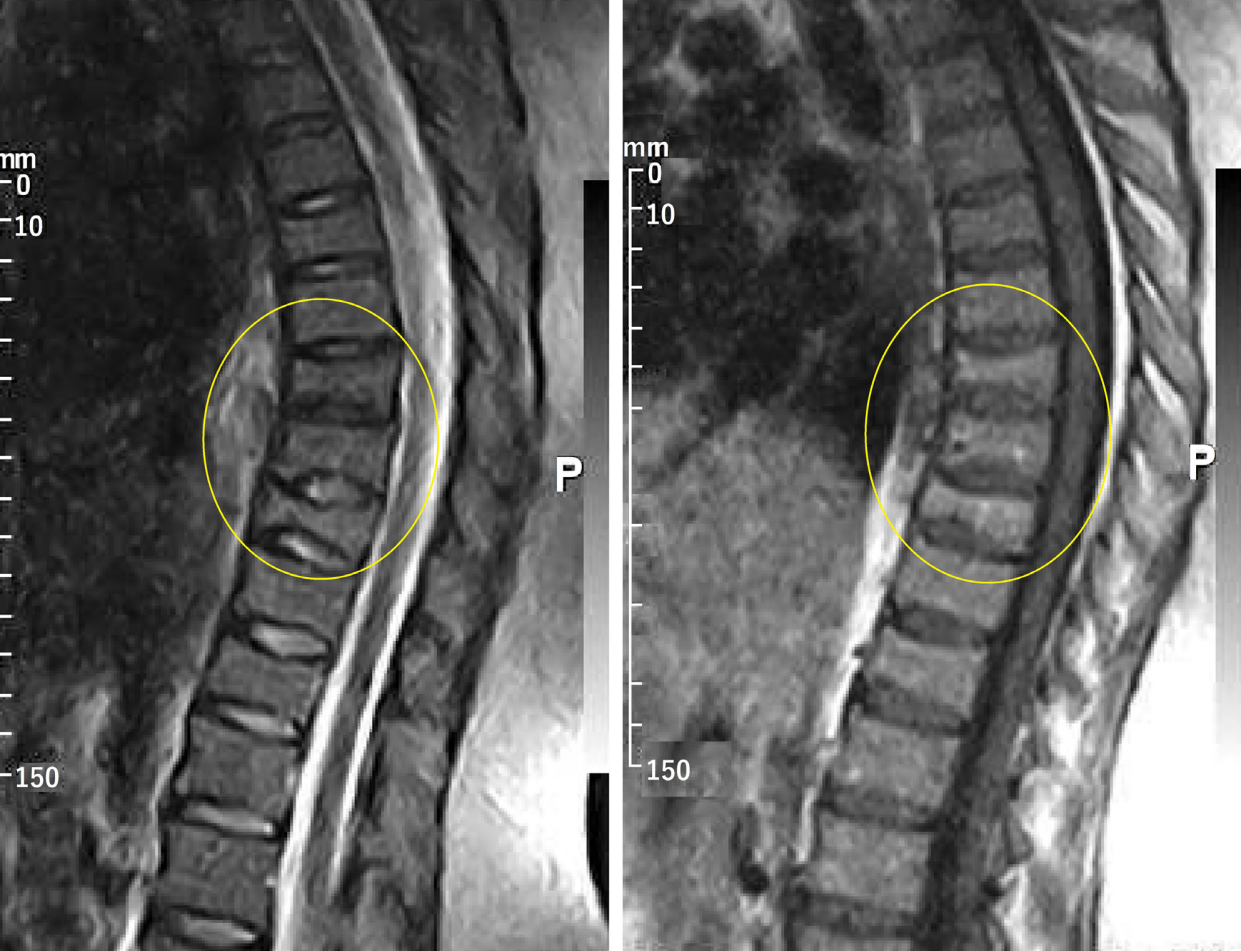
MRI of the thoracic vertebrae. Collapse is seen in T9‐T12 vertebrae.

**FIGURE 2:**
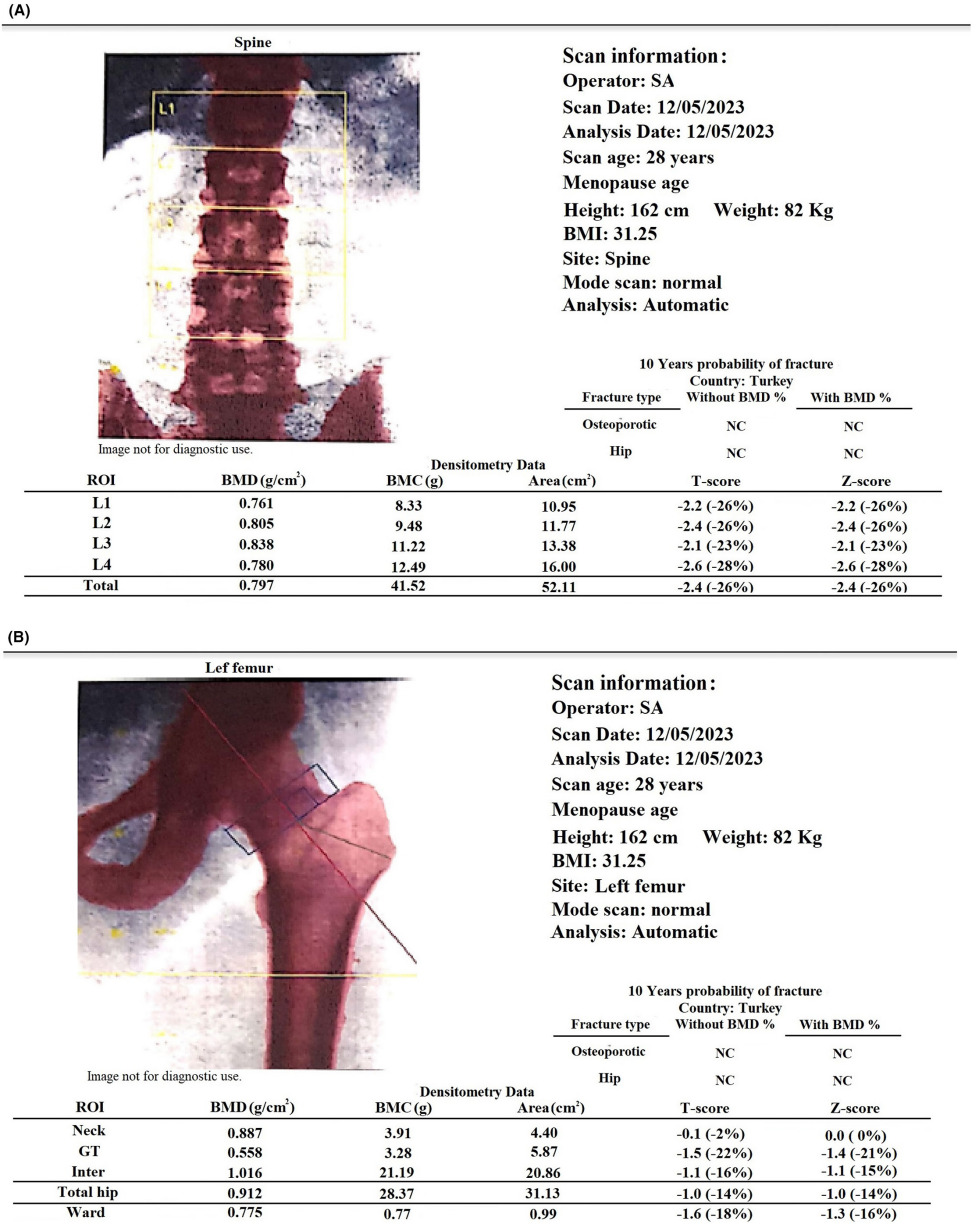
Dual‐energy x‐ray absorptiometry (DEXA) scan of bone mineral density (BMD) of the lumbar (A) and femur (B).

A 28‐year‐old woman with osteoporotic fractures of thoracic vertebrae was referred to us 2 weeks after delivery. She was a nulliparous woman who had been infertile for 3 years because of her husband's azoospermia. She had been trying to conceive through IVF and had a cycle of superovulation with gonadotropin‐releasing hormone (GnRH). Ovulation was induced through the administration of 100 mg of oral clomiphene daily for 5 days, followed by injectable gonadotropins from the 2nd to the 14th day of the cycle. Subsequently, ovulation was stimulated using 1 mg of subcutaneous leuprolide acetate, supplemented by a rescue dose of human chorionic gonadotropin (1500 IU intramuscularly). Post‐conception, the patient was prescribed enoxaparin at a dosage of 40 mg twice daily up until the 39th week of gestation, and aspirin at a dosage of 80 mg daily until the 36th week of gestation. She had a balanced diet and normal activity and never experienced pregnancy complications such as nausea or preeclampsia. In the 39th week of pregnancy, she gave birth to a healthy newborn by cesarean section. Two days after delivery, she experienced severe back pain. She had no history of trauma. Due to pain that could not be improved with analgesics and severe tenderness in the thoracic spine, magnetic resonance imaging (MRI) of the spine was performed. Wedge‐shaped fractures of T9‐T12 vertebrae were reported in MRI (Figure 1). In dual‐energy x‐ray absorptiometry (DXA, Stratus), BMD of L1‐4 and femoral neck was 0.797 (total) and 0.887 g/cm^2^, respectively (Figure 2). The reported Z‐score for L1‐4 and femoral neck was −2.4 (total) and 0.0, respectively. She was referred to our clinic 2 weeks after the onset of back pain. She was a nonsmoker and had no other significant past medical history. There was no history of osteoporosis and low‐trauma fracture in her parents. On examination, vital signs were normal. Height, weight, and BMI were 162 cm, 82 kg, and 31.25 kg/m^2^, respectively. Tenderness existed on the percussion of her lower thoracic spine. There was a severe limitation in all spinal movements. The laboratory tests were within the normal range (Table 1).

**TABLE 1:** Serum laboratory tests.

Lab test (unit)	Result (normal range)
Hemoglobin (g/L)	12.1 (12–14.5)
Leukocyte count (per μL)	8200 (4000–10,000)
ESR (mm/h)	31 (0–30)
CRP (mg/L)	7.3 (0–6)
BUN (mg/dL)	18 (7–20)
Creatinine (mg/dL)	0.8 (0.6–1.1)
Urine analysis	Normal
AST (g/dL)	32 (8–35)
ALT (g/dL)	28 (8–35)
Alkaline phosphatase (U/L)	192 (100–340)
Albumin (g/dL)	4.1 (3.4–5.4)
Calcium (mg/dl)	10 (8.6–11)
Phosphorus (md/dl)	4.3 (2.6–4.5)
24 h urine calcium (mg/dL)	152 (100–250)
iPTH (pg/ml)	43.2 (16–46)
25‐hydroxy vitamin D3 (ng/ml)	29.1 (30–100)
TSH (μIU/mL)	2.9 (0.35–5.58)
Prolactin (mIU/L)	210.4 (132–498)

Abbreviations: ALP, alkaline phosphatase; ALT, aspartate alanine transferase; AST, aspartate aminotransferase; BUN, blood urea nitrogen; CRP, C‐reactive protein; ESR, erythrocyte sedimentation rete; iPTH, intact parathyroid hormone; TSH, thyroid‐stimulating hormone.

## 3 METHODS (DIFFERENTIAL DIAGNOSIS, INVESTIGATIONS, AND TREATMENT)

Based on the clinical manifestations, osteoporotic spine fracture, results of BMD, and exclusion of other causes of osteoporosis, the patient was diagnosed with PAO. Breastfeeding was stopped and treatment with calcium 1000 mg/day, vitamin D3 1000 IU/day, alendronate 70 mg/week, naproxen 250–500 mg/d, and nortriptyline 20 mg/d were started.

## 4 CONCLUSION AND RESULTS

Two weeks later, an improvement in the patient's pain and mobility was observed, and after 6 weeks, the patient was able to stand up and perform her daily activities.

## 5 DISCUSSION

In February 2023, there were 80 reported cases of PAO on MEDLINE/PubMed. We presented a case of PAO in a woman conceived with IVF. The presented case did not have any known risk factors for PAO except enoxaparin use. Although heparin has mainly been implicated as a risk factor for PAO, several animal studies have shown that low molecular heparins (LMWHs) like enoxaparin dose dependently inhibit osteoblasts proliferation and impairs the mechanical properties of bone.^3^, ^4^ There are reports of PAO in women treated with enoxaparin.^5^, ^6^ In contrast, an observational cohort study of 152 women who had received LMWH during pregnancy concluded that LMWH use was not associated with a subsequent decrease in BMD and osteoporotic fractures.^7^ In the largest case series of PAO, which compared 102 PAO patients with 102 healthy controls, LMWHs were not introduced as a risk factor.^8^ It has been hypothesized that repeated ovulation on ovaries may affect the structure and function of the ovaries and lead to ovarian aging.^9^ A decrease in sex hormone production caused by ovarian aging may affect the cardiovascular system, bone density, mental health, and sexual function in women.^10^ Few studies have focused on the long‐term effects of ovarian hyperstimulation on health outcomes, particularly bone density and structure. Zhang et al. showed that bone density significantly reduces in mice exposed to repeated superovulation.^11^ They suggested that repeated superovulation may increase the risk of osteoporosis. In 2018, we presented a case of PAO that was a candidate for IVF and had received GnRH.^12^ However, she got pregnant after one month without IVF. Treatment with GnRH may be a risk factor for PAO due to the hypoestrogenic state it induces, accelerating bone loss.^13^ Moreover, studies collectively suggest that pregnancy‐induced hormonal changes lead to ligament laxity, potentially causing stress fractures in the pelvic region due to altered connective tissue and increased muscular stress, especially during delivery.^14^, ^15^, ^16^

In conclusion, although PAO is not a hypoestrogenic state and the etiology of PAO is not known, by reporting this case, we hypothesized that IVF may be an etiologic factor for PAO due to the use of GnRH agonists and ovarian hyperstimulation.

## AUTHOR CONTRIBUTIONS

**Mehran Rahimi:** Data curation; software; writing – original draft. **Sara Daneshvar:** Data curation; software; writing – original draft. **Alireza Khabbazi:** Conceptualization; supervision; writing – review and editing.

## FUNDING INFORMATION

None.

## CONFLICT OF INTEREST STATEMENT

The authors have no conflict of interest to declare.

## ETHICS STATEMENT

Written consent was provided by the patient.

## CONSENT

Written informed consent was obtained from the patient for publication of their case and any accompanying images.

## Data Availability

All data related to the patient are presented in the text and figures of this case report.
